# Supplementary material for: New Genes in the Drosophila Y Chromosome: Lessons from D. willistoni
Source: Genes (Basel). 2021 Nov 18;12(11):1815. doi: 10.3390/genes12111815 (PMC8623413; doi:10.3390/genes12111815)
Supplement: Supplementary file 1 [file genes-12-01815-s001.zip › genes-1429201-supplementary-final.pdf]

genes

MDPI

Article

New genes in the *Drosophila* Y chromosome: lessons from *D. willistoni*

João Ricchio [1], Fabiana Uno [1] and A. Bernardo Carvalho [1,*]

[1] Departamento de Genética, Instituto de Biologia, Universidade Federal do Rio de Janeiro, Brazil; rosaricchio@gmail.com (J.R.); fabi.uno@gmail.com (F.U.); bernardo1963@gmail.com (A.B.C.)

* Correspondence: bernardo1963@gmail.com

Supplementary Materials

The Supplementary Material is composed of:

a) This file, containing text, three Supplementary Figures and eigth Supplementary Tables (pdf).
b) The spreadsheet file YGS_results.xlsx (Excel).
c) The wilYgenes_25oct2021_cds.fasta file, containing the sequence of the annotated genes (plain text).
d) The R script file Poisson_regression.R (plain text).
e) The R data file AF_vir_mel_wil_data.txt (plain text).
f) The R data file AF_vir_mel_wil_conservative_data.txt (plain text).

Annotation of *D. willistoni* Y-linked genes

Here we detail the annotation of the 14 new Y-linked genes we found. As we commented in the main text at least a small part of each of them has been previously annotated using computational pipelines, and we choose to keep the original gene names. Given these previous efforts, it is desirable to clarify here the contributions of the present manuscript. Currently there are three computational annotations. The first was done when the *D. willistoni* genome was sequenced in ~2005 [1], and later updated by FlyBase. Around 2010 FlyBase stopped updating the gene annotations of "non-melanogaster species", and NCBI assumed this task. Finally, Yang *et al.* [2] reannotated eight *Drosophila* genomes, including *D. willistoni*. The last two annotation efforts made extensive use of Illumina RNAseq data which was not available when the first annotation was done, and in general are more complete and precise. There are two broad limitations in these previous annotation efforts. First, none of the 14 genes was recognized before as a Y-linked gene. Second, in the three previous annotations the sequence of the 14 genes is incomplete for more than half of the genes (Table S7), partly because these annotations were completely automatic, *i.e.*, there was no manual curation. We addressed the above problems as follows.

Regarding Y-linkage, as described in the main text, we used a computational method [3] to detect candidate Y-linked sequences, and experimentally confirmed it with PCR for all 14 genes.

The second problem is quite common in Y-linked genes. The culprit is the huge size of the introns, which causes assembly fragmentation: exons of the same gene end up scattered in different scaffolds. All three computational pipelines mentioned above are strictly based on the genome sequence, even when they use RNAseq data. Hence, the genes that were fragmented in the genome assembly are also fragmented in the annotation. For example, the exons of the complete *GK27406* gene are scattered in three scaffolds in the reference Sanger genome assembly (CH962205, CH968469, and CH964169), and each part

was annotated as a different gene in Clark *et al.* [1] (*GK18657*, *GK27211*, and *GK27406*, respectively). A similar result is present in the annotation done by Yang *et al.* [2] , and NCBI annotated just one of the partial sequences. Besides the fragmentation issue, some exons are missing in the genome assembly, and were not annotated. We addressed these problems by manually annotating these genes using TblastN (which frequently retrieves the scattered exons), followed by RT-PCR or *de novo* assembly of RNAseq reads to demonstrate that the scattered exons belong to the same transcription unit, and to close the gaps, as described in ref. [4]. The above methods work well for single-copy genes, or genes with two copies. The annotation of multi-copy genes presented some specific challenges, as detailed below.

Annotation of multicopy genes *GK20618* / *GK20619*, and *GK18510*.

GK20618 / *GK20619* encodes a β6 subunit of the proteasome. *D. melanogaster* harbors only one copy of the *Prosβ6* gene, which has widespread expression, whereas there are seven paralogs in *D. willistoni* (Fig S1 and Table S3). Three of them are autosomal: *GK20318* (which has widespread expression) and the testis-specific *GK16137* and *YOgnWI012342*. The duplication of proteasome subunit genes originating testis-specific paralogs is common in *Drosophila* species, and was noticed by Belote and Zhong [5] in many of them, including *D. willistoni*. In *D. willistoni* the testis-specific *YOgnWI012342* further duplicated to the Y chromosome and once there, additional duplications originated a total of four genes. They are all very similar (98% identity between the Y-linked genes and the autosomal *YOgnWI012342*; 99% among the Y-linked genes), which may cause misassemblies. Indeed, the four Y-linked copies are present only in the PacBio assembly. The Sanger assembly, which is the genome reference used in the available gene annotations [1,2], contains only two Y-linked copies, *GK20618* and *GK20619*, which are identical to two PacBio copies. Finally, there is one Y-linked pseudogene in the Sanger assembly (*GK22295*) and three in the PacBio assembly. Even though we cannot directly compare these results with the ones obtained with the Nanopore assembly, as it came from a different strain, we saw a similar pattern there: three functional Y-linked copies and two Y-linked pseudogenes. While we are fairly confident that the *D. willistoni* Y-linked copies of *Prosβ6* are functional, the above results must be taken cautiously. Highly similar gene copies are prone to misassemblies with smaller reads such as Illumina and Sanger, but they can also occur with long reads [6]. All evidence of gene functionality (intactness of coding region; gene expression) relies on the correctness of the assembled genome, and more detailed studies are needed to confirm that (*e.g.*, ref. [6]).

The second multi-copy gene with difficult annotation was *GK18510*, which encodes a sperm protamine. It looks simple in the Sanger assembly: two Y-linked copies (scaffolds CH971205 and CH968555), and one annotated transcript (*GK18510*, from the first scaffold). But there are ~ 20 copies in the PacBio and Nanopore assemblies, some complete, some fragmented, and none of them perfectly match the *GK18510* sequence. Hence, the current sequence of *GK18510* most likely represents collapsed copies of the Y-linked sequences, *i.e.*, it is misassembled. Given the many copies and their fragmented state, the PacBio and Nanopore assemblies may also have misassemblies in this multicopy gene. All the caveats mentioned above for the *Prosβ6* genes apply much more strongly to *GK18510*, and its classification as a functional gene should be seen as tentative.

Statistical test of gene gains and gene losses

Here we investigate the rates of gene gain and gene loss in the *D. willistoni* lineage, and compare it with previous results from two other species. We did this with the "Assumption-free" method described in ref. [3], which can only be used with Y chromosomes with well known gene content, such that both gene losses and gene gains were identified. Koerich *et al.* 2008 [7] and Carvalho and Clark 2013 [3] also used two other methods ("Homogeneous Gain Loss" and “Approximate Bayesian Computation”) which are specially

helpful when Y-linked gene content is well known in only one species. As we now have three species with well known gene content of the Y (*D. virilis* [3], *D. melanogaster* [7], and *D. willistoni* (this paper)), we applied the "Assumption-free" method, which is simpler. We have not included *D. pseudoobscura* and related species because in this lineage the entire Y chromosome became part of an autosome [8]. This should not be confounded with an event of gene loss, in the same way that the neo-Y formation in *D. miranda* [9] should not be confounded with gene gain events. These chromosome-wide events have different causes and consequences from the individual gene gains and losses, which we are dealing with in the current paper.

The gene gains and losses in the three species phylogeny are shown in Figure S3, and the divergence times were taken from ref. [10]. There are fairly large uncertainties in these divergence times, but we are interested in the gain-loss ratio, which is not affected by these uncertainties (they cancel out in the gain-loss ratio; see ref. [7] for details). We implemented the statistical procedures, which are based on Poisson regression, in R language [11]. They test the null hypothesis of a gain-loss ratio of 1 (see refs. [3,7] for details). The R script and the input data files are in the Supplementary files Poisson_*regression.R* and *AF_vir_mel__wil_data.txt*, respectively.

We found a gene gain / gene loss ratio of 25.0 (P = 0.002, Poisson regression; 95% confidence interval: 3.4 - 184.5). Previous studies have shown that the gain-loss ratio is significantly larger than 1 in both *D. melanogaster* and *D. virilis* branches [3,7], and the same obviously is true in the *D. willistoni* branch, which has more gene gains than these species, and no gene loss. The Poisson regression also tests the goodness-of-fit of the model to the data, which here tests the heterogeneity of gain-loss ratio in the branches shown in Figure S3. We found a statistically significant heterogeneity (P = 0.006). Thus, it seems that the preponderance of gene gains over gene losses is a general phenomenon in *Drosophila*, and that its magnitude is variable across the phylogeny.

The same qualitative results are obtained if we adopt a more conservative interpretation of the *D. willistoni* results, by excluding the very recent three gene gains which have nearly identical copies in the autosomes (the genes are *GK18510*, *GK20591*, and *CG34175*), on the grounds that it is less certain that they will not become pseudogenes. In this case we found a gene gain / gene loss ratio of 21.0 (P = 0.003, Poisson regression; 95% confidence interval: 2.8 - 156.1), and a statistically significant heterogeneity (P = 0.02).

Table S1. Illumina datasets. All reads came from the reference strain of *D. willistoni* (14030-0811.24).

Accession	Source
SRR9426110	adult male DNA
SRR9426117	adult female DNA
SRR15992681, SRR15992682	adult male accessory gland RNA
SRR15992683, SRR15992684	adult female ovary RNA
SRR15992685, SRR15992686	adult male testis RNA
SRR5639520, SRR7243441	adult male whole body RNA
SRR5639514, SRR7243435	adult male thorax RNA
SRR5639502, SRR7243423	adult male head RNA
SRR5639517, SRR7243438	adult female whole body RNA
SRR5639511, SRR7243432	adult female thorax RNA
SRR5639499, SRR7243420	adult female head RNA

Table S2. PCR primers used to test for Y-linkage in *D. willistoni* and other species.

Target gene	Forward primer	Reverse primer
CG34277	RCCC_F1 CAATCTTCTGGAAGACCAATGATGMRNTGYWSNTG	AVVA_R1 ACCTCCTATACCTTGTATTTTACCAMGNYGNTGNCG
CG34277	CG34277_F10 ACCAAGGATTCCGCTGCCATTA	CG34277_R10 GTGTGAAGCGAGGATGAT
CG6052	VWDY_F1 TGTTCTTATTGGATGTCTCARTGGGTNTGGGAYTA	MIAI_R1 GGTTATGTAAAATAAACAGGTCTTTAGTAHTANCGHTA
CG6052	VVDS_F2 GAAGAACGAAATCAAATGCCAYTNRTNGTNGA	MIAI_R2 GGTTATGTAAAATAAACAGGTCTTTAGTAHTANCGHTA
CG6052	CG6052_F10 GCCATTGGTAGTGGACTCGATTAGTA	CG6052_R10 ATTTTCAGTATTAATCCCTTGGAGTAA
CG32650	EQNH_F1 TGGATTCATTCTAAATATCTACAAATGGARCANAAYCA	FDWD_R1 TAAGCTAAAACAGCTAGGGTYAGYTT
CG32650	CG32650_F10 AGTATGTTTTCGCAATTTAGGAGA3	CG32650_R10 CAAGAAACAACGGTGATGGT
Ran-like	RAN_F10 AGTGTTTATTGCTTGGAGA	RAN_R10 AGGAGAAGTATAGGCCACATCAAG
Pzl	PZL_F10 AAACTTTGCTGATTGGATG	PZL_R10 GGGGGAAAGTTCAATAAGT
CG15580	CG15580_F10 GGGTGTTAGCCTCAAGTATGTG	CG15580_R10 CACACCTCATTGGCCACTATTTCT
CG34175	CG34175_F10 TAATCCATTTTACATAGGTCCCAATCCACCGCAAT	CG34175_R10 TTACGTTCTGGCTACTTGGACGGCAATTGTAA
CG14740	CG14740_F10 TATATTTTACCGGGGCATGACGCTCAAGGAT	CG14740-R10 ACTCTCGAGATCCAGCACCGTCCTTAAATGCATTG
CG14718	CG14718-F10 TTGTTATTGGGCAGAAGCAGCTACTGTGGATTCC	CG14718-R10 ACTCAGACGCATGCCCAGCACAAAGATCGC
CG6888	CG6888-F10 CTAACCGAGTTTCGTGGTCGTTATGTGGTGC	CG6888-R10 TTATTGAGCATTTTTAAAGTAATCATCT
CG6888	CG6888-F11 ACATGCGTTCAGCGATCGGGCCCAGGAATTCA	CG6888-R11 ACCGAACTCATCGCTGAACTGGAACGCCTGA
CG10588	CG10588_F10 SEQUENCE GAAATTTCAATACCGCACTAA	CG10588_R10 ATCCATTAAACATTCAGTCC
CG10588	CG10588_WRKY_F1 CATGAAGCTCTACATGATTTTTGGMRNAARTA	CG10588_VLPP_R1 CCAAATCAAGCTATTACTTTAAAGTAACSNCCNTYNTG
CG10588	CG10588_DNVQ_F2 TTTCAAGTTGAACTACCATTTATGGAYAAYGYNCA	CG10588_TLHW_R2 CCAAAAGGAACAACTACGGTYWYNTYNCA
CG13539	CG13539_DDRL_F1 ATTCATGGTCATCTACAACTAATGGAYGANMGNYT	CG13539_FNCM_R1 CTTGATCTTTTAATTTACCACGTAAGTHYGTYAAYTT
CG13539	CG13539_NCST_F2 CTACAAACTATGAAAATTATTAAACGAATGAAYTGYWSNAC	CG13539_TASV_R2 TTGTAACTCAATCAAATCGTCTGGTTTRNSWNCGNCA
CG13539	CG13539_MKRH_F3 AGATGCTATTCCAGATATTTATTTTCATCTAHTNAARMGNCA	CG13539_PQYY_R3 AGGTCCACCTATTAGAGGTTTTATYATRACNCC
CG13539	CG13539_F10 AGAAAATTGCAAAAACTGGA	CG13539_R10 TTTTACGAGACATGCTACAATC

Table S3. Expression (in fpkm) of *Prosβ6* gene family in *D. willistoni*.

**Sample**	***GK20318***	***GK16137***	***YOgnWI012342***	***GK20618***	***GK20619***	***GK22295***
male whole body	242.8	15.6	33.2	52.5	48.5	0.0
testis	329.5	103.5	232.7	621.2	562.9	9.1
accessory gland	335.7	0.0	0.3	0.8	0.1	0.0
male thorax	108.7	0.1	0.7	1.7	0.6	0.0
male head	156.6	0.2	1.2	0.5	0.0	0.3
female whole body	453.6	0.0	0.0	0.0	0.0	0.0
ovary	459.9	0.0	0.1	0.0	0.0	0.0
female thorax	117.8	0.0	0.0	0.0	0.0	0.0
female head	152.6	0.0	0.0	0.0	0.0	0.0

Table S4. Expression (in fpkm) of Y-linked genes and their close autosomal paralogs.

**Sample**	**GK18510 (Y)**	**GK18077 (A)**	**GK20591 (Y)**	**GK19651 (A)**	**YOgnWI018045 (Y)**	**GK14595 (A)**
male whole body	857.2	612.5	38.9	31.2	23.4	46.6
testis	8218.6	5664.0	390.9	209.8	209.6	310.1
accessory gland	9.6	6.4	0.3	0.3	0.5	0.0
male thorax	14.0	8.9	0.6	0.1	0.0	0.0
male head	20.1	12.2	0.2	1.6	0.5	1.4
female whole body	0.0	0.0	0.0	0.0	0.0	0.4
ovary	0.4	0.3	0.1	0.0	0.0	0.0
female thorax	0.0	0.0	0.0	0.0	0.0	0.0
female head	0.0	0.0	0.0	0.0	0.0	0.0

Table S5. *D. willistoni* Y-linked pseudogenes.

***D. willistoni* functional gene**		***D. willistoni* Y-linked pseudogene**		***D. melanogaster* ortholog** [b]	
**Name**	**Scaffold** [a]	**Scaffold** [1]	**Evidence of pseudogenization**	**Name**	**Expression**
GK20831	4510	1610	no expression	*Roc1a*	widespread
GK20808	4510	744	no expression	*CG5819*	widespread
GK15752	4514	2710	no expression	*CG8701*	testis
GK15702	4514	4162	no expression	*CG16926*	widespread
GK15705	4514	1375/4911	no expression	*TBCB*	embryo / larva
GK15704	4514	4/3948	no expression	*Oseg6*	embryo / larva
GK15700	4514	647	no expression	*Ir56d*	widespread
GK15874	4514	4635/2143/4911	deletions	*par-1*	pupa
GK15875	4514	4911	deletions	*Rep*	widespread
GK15703	4514	9519	deletions	*hpo*	widespread
GK14599/GK14600	4521	10303/12259/13106	stop codons	*Lrr47*	ovary
GK18073	4577	4598/1443/778	stop codons	*CG42313*	embryo
GK18305	4577	4453/2052	no expression	*CG3491*	testis
GK18085	4577	11518	deletions	*CG32164*	widespread
GK18304	4577	306/2402	no expression	*CG4701*	testis
YOgnWI012342	4577	4749	no expression	*Prosβ6*	widespread
GK18076	4577	338	deletions	*CG33308*	testis
YOgnWI012332	4577	4687	stop codons	*CG33309*	testis
GK27571	4585	669/1241/6417	no expression	*CG18109*	testis
GK11576	4902	14514	no expression	*CG15186*	widespread
GK25126	4909	3178/14016	no expression	*CG9940*	widespread
GK24923	4909	1787/4784	no expression	*CG4078*	widespread
GK22673	4921	4544	deletions	*CG1458*	widespread
GK22587	4921	3711/2127	no expression	*CG32625*	embryo
GK22615	4921	4753	no expression	*bnk*	widespread
GK22587	4921	402	no expression	*CG34283*	testis
GK28182	4943	2273/676/795	no expression	*Osi9*	embryo / larva
GK13038	4943	10790/2374/14678	no expression	*Osi8*	pupa
GK13036	4943	1766/1205/5776	no expression	*Osi6*	embryo / pupa
GK14206	4943	10032/6949	no expression	*Osi11*	pupa
GK13044	4943	1946/4878/5852	no expression	*Osi12*	widespread
GK13035	4943	5128/13128	no expression	*Osi5*	widespread
GK13153	4943	3173/3481	no expression	*Ppi1*	testis
GK13155	4943	11089/14856	no expression	*Irk2*	larva
GK13040	4943	1228/2085	no expression	*Osi10*	larva/pupa
GK13026	4943	287/3758/4722	no expression	*Vha14-2*	testis
GK18942	4943	4666/4685/429	no expression	*CG10177*	testis
GK13030	4943	4938	deletions	*CG16898*<br>*CG33301*	digestive system
GK13037	4943	13467	deletions	*Osi7*	embryo/ pupa
GK26902	4943	5712	deletions	*CG7208*<br>*CG31948*	gonads
GK28338	4943	3990	out of frame indel	*CG7208*<br>*CG31948*	gonads
GK18354	4945	6321	deletions	*E23*	larva
YOgnWI027949	4945	8817	no expression	*CG34394*	larva
GK18364	4945	7938	stop codons	*l(2)gd1*	widespread
GK18206	4963	4425/4684	no expression	*CG10934*	testis

[a] *D. willistoni* scaffold names are abridged as follows. "CH960481 scf2_1100000001122" abridged as "1122".

[b] Orthology information taken from FlyBase [12].

Table S6. Time of acquisition of genes that retained the autosomal or X-linked original copy. PCR tests are not safe in these cases, so we relied on BlastN searches (see Material and Methods for details). We found that the last three genes (*GK20591*, *YOgnWI018045*, and *GK20618*) were duplicated to the *D. willistoni* Y chromosome after the split between this species and *D. paulistorum* / *D. equinoxialis* (see Figure 3 for the phylogeny of these species). The result of *GK18510* is inconclusive: under a single-event scenario the sister species *D. paulistorum* and *D. equinoxialis* should have been identical, either having many copies (indicating a duplication to the Y chromosome before their split from *D. willistoni*) or only one copy (indicating a duplication after this split). Hence, we can only say that the duplication happened after the split between *D. tropicalis* and the ancestor of the other species.

Gene	*D. melanogaster* ortholog	Number of blastN full copies [a]					
		D. willistoni Sanger	*D. willistoni* PacBio	*D. willistoni* Nanopore	*D. paulistorum* [b]	*D. equinoxialis*	*D. tropicalis*
GK18510	*ProtA*	~ 10	~ 10	~ 10	~ 10	1	1
GK20591	*CG6888*	2	2	2	1	1	1
YOgnWI018045	*CG34175*	2	1 [c]	2	1	1	1
GK20618	*Prosβ6*	5	8	6	1	1	2 [d]

[a] BlastN search using the *D. willistoni* Y-linked genes as queries and the listed genomes as the databases.

[b] The two strains of *D. paulistorum* [13] produced identical results.

[c] This result in unexpected, because the Sanger and PacBio assemblies came from the same strain (but with a ~15 years interval). The discrepancy may be caused by an assembly failure in PacBio, or mutation in the *D. willistoni* stock.

[d] Possibly the result of an independent duplication of *Prosβ6* in the *D. tropicalis* lineage [5].

Table S7. *D. willistoni* Y-linked genes as represented in successive annotation efforts. Only matches to our final annotation (Supplementary File wilYgenes_25oct2021_cds.fasta) that have 100% identity and 100% coverage of are shown.

Gene	*D. melanogaster* ortholog	Copies [a]	Clark *et al.* 2007 [b]	Yang et al. 2018 [c]	NCBI [d]	Testis RNAseq [e]	Source of final annotation [f]
GK21041	*CG18155*	1 Y		*YOtrWI000247*	XM_023179564.1	contig5626	*YOtrWI000247*
GK20609	*CG15580*	1 Y	*GK20609*			contig3806	*GK20609*
GK13929	*CG10588*	1 Y	*GK13929*		XM_023177135.1	contig1847	*GK13929*
GK28041	*CG32650*	1 Y		*YOtrWI024144*	XM_015177028.2	contig10096	*YOtrWI024144*
GK27472	*CG13539*	1 Y				contig11488	contig11488
YOgnWI030283	*CG34277*	1 Y				contig10961	contig10961
GK21220	*CG6052*	1 Y				contig489	contig489
YOgnWI000172	*CG14339*	1 Y				contig2432	contig2432
GK27406	*Piezo-like*	1 Y				contig223	contig223
GK28211	*Ran-like*	1 Y				contig9500	contig9500
GK18510	*ProtA*	10 Y, 1 A	*GK18510*	*YOtrWI030169*	XM_002075936.3		*GK18510*
GK20591	*CG6888*	1 Y, 1 A	*GK20591*	*YOtrWI024161*	XM_002072443.2	contig14861	*GK20591*
YOgnWI018045	*CG34175*	1 Y, 1 A		*YOtrWI018045*			*YOtrWI018045*
GK20619	*Prosβ6*	4 Y, 1 A	*GK20619*	*YOtrWI000244*	XM_002060798.1		*GK20619*
wilProsB6_Y2	*Prosβ6*	4 Y, 1 A					PacBio assembly
wilProsB6_Y3	*Prosβ6*	4 Y, 1 A					PacBio assembly
wilProsB6_Y4	*Prosβ6*	4 Y, 1 A					PacBio assembly

[a] The number of copies in the *D. willistoni* genome. The values for *GK18510* and *wilProsB6_Y* genes are approximate.

[b] Release 1.3, obtained from FlyBase (http://ftp.flybase.net/genomes/dwil/dwil_r1.3_FB2008_07/fasta/dwil-all-CDS-r1.3.fasta.gz).

[c] Yang *et al.* [2] reported full transcript sequences as gff files (*i.e.*, with 5' and 3' UTRs). We annotated the coding sequences (CDS) comparing them to the *D. melanogaster* orthologs.

[d] NCBI annotations are periodically updated. The data shown was accessed in October 5th 2021.

[e] Accession GJOF01000000. The testis transcriptome contains full transcripts (*i.e.*, with 5' and 3' UTRs). We annotated the coding sequences (CDS) comparing them to the *D. melanogaster* orthologs.

[f] For the sake of simplicity we collated all final cds sequences in a single file (Supplementary File wilYgenes_25oct2021_cds.fasta).

Table S8. Gene gains and losses in the Y chromosomes of three *Drosophila* species. See Figure S3 for the phylogenetic context.

Branch	Branch length (Myr) [a]	Gene losses	Gene gains [b]	Source
A-B	0.7	-	*ARY, CCY*	[7]
B-wil	62.2	-	see Table 1 (14 genes)	This paper
B-mel	62.2	*JY-alpha*	*WDY, kl-5, Pp1-Y1, Pp1-Y2, FDY*	[3,7]
A-vir	62.9	-	*kl-5, GJ19835, CG11719, CG2964*	[3]

[a] Branch lengths were taken from ref. [10].

[b] The *kl-5* gene was independently gained twice (see Figure 1 of ref. [7]).

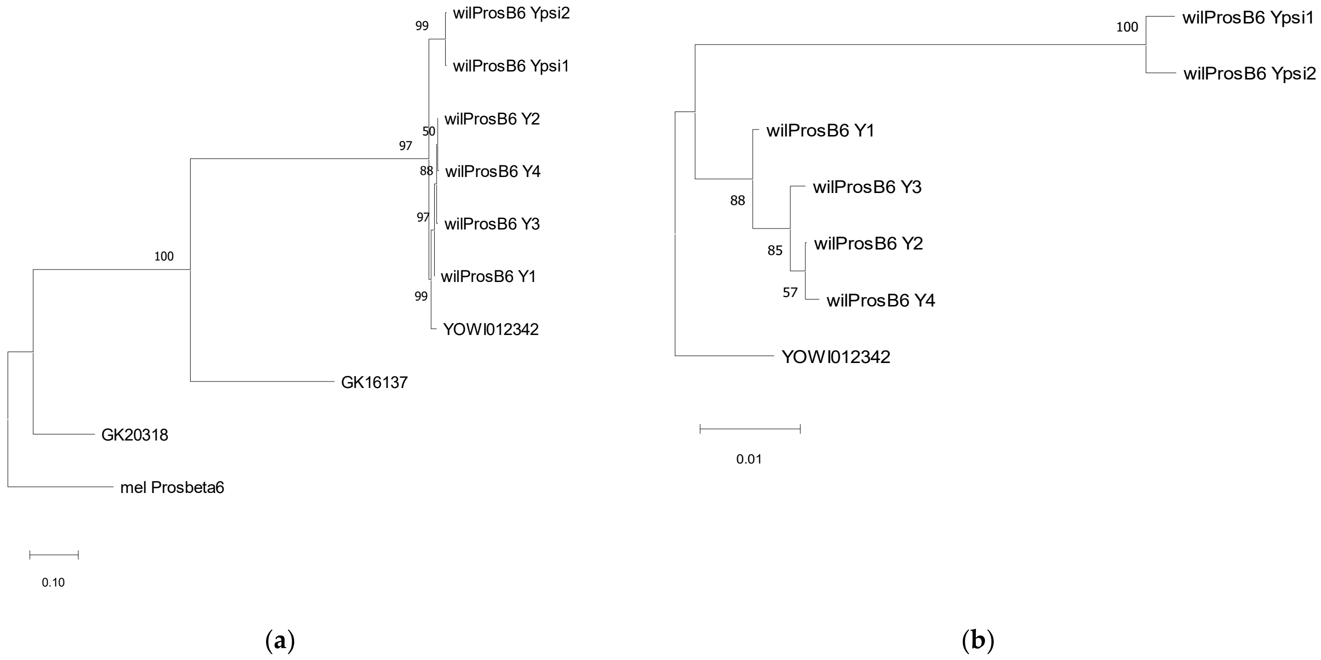

Figure S1. Phylogeny of the *Prosβ6* gene family in *D. willistoni*. (**a**) Phylogeny including all *D. willistoni* genes, rooted in *D. melanogaster*. (**b**) Phylogeny including only the *D. willistoni* Y-linked genes, rooted in the autosomal gene that originated them (*YOgnWI012342*). *D. melanogaster* harbors only one *Prosβ6* gene, which has widespread expression. In contrast, there are seven genes in *D. willistoni*, plus two pseudogenes. The *GK20318* gene has widespread expression, and all others are testis-specific. *GK16137* and *YOgnWI012342* are autosomal; the latter was copied to the Y chromosome as part of a segmental duplication. Once there, it generated four functional genes (labeled as *wilProsB6_Y1* to *wilProsB6_Y4*, and two pseudogenes (*wilProsB6_Ypsi1* and *wilProsB6_Ypsi2*). These genes and pseudogenes were annotated from the PacBio assembly because the reference Sanger assembly apparently collapsed several copies, and contains only two functional genes: *GK20618* (partial sequence, 100% identity with *wilProsB6_Y2*) and *GK20619* (100% identical to *wilProsB6_Y1*). It also includes one pseudogene, *GK22295*, which is a partial sequence with 99% identity with the two pseudogenes annotated in the PacBio assembly. Phylogenies were inferred with the Maximum Likelihood method on the nucleotide sequences and the Tamura 3-parameter model with rate differences among sites modeled by a Gamma distribution. Analyses were performed in MEGA11 [14].

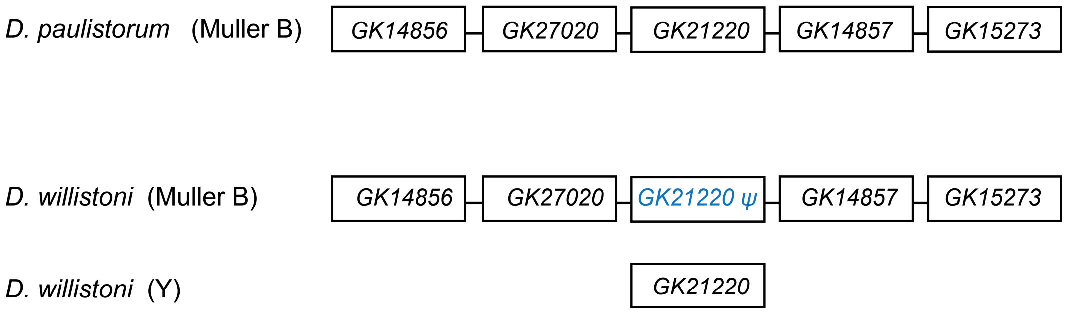

Figure S2. Ancestral autosomal location of the Y-linked gene *GK21220*. BlastN searches with the Y-linked gene *GK21220* showed the Y-linked scaffolds (as expected) and a very similar 118 bp match in the autosomal scaffold CH963850 (coordinates 5158275- 5158392). The corresponding region in the *D. paulistorum* assembly is autosomal (as expected) and contains the functional ortholog of the *GK21220* gene. Hence, the 118 bp region of scaffold CH963850 in the *D. willistoni* genome is a remnant of the original *GK21220* gene, which degenerated after its duplication to the Y. We could not find any sign of the flanking genes in the *D. willistoni* Y, either because they were not copied to the Y, or had degenerated beyond recognition.

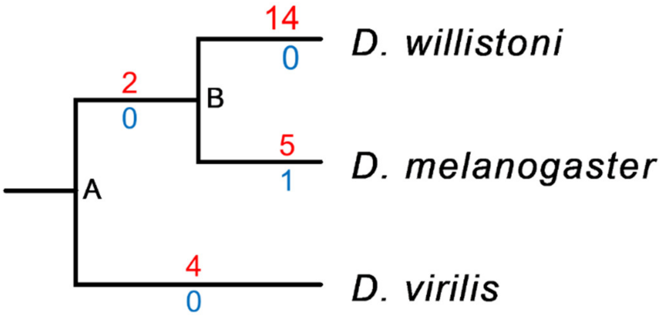

Figure S3. Gene gains and losses in Y chromosomes of three *Drosophila* species. The numbers of gene gains are shown above the respective branches in red, and the gene losses are shown in blue. See Table S8 for the list of genes. Nodes are labeled in black.

References (Supplementary Material)

1. Clark, A.G.; Eisen, M.B.; Smith, D.R.; Bergman, C.M.; Oliver, B.; Markow, T.A.; Kaufman, T.C.; Kellis, M.; Gelbart, W.; Iyer, V.N.; et al. Evolution of genes and genomes on the *Drosophila* phylogeny. *Nature* **2007**, *450*, 203-218, doi:10.1038/nature06341.
2. Yang, H.; Jaime, M.; Polihronakis, M.; Kanegawa, K.; Markow, T.; Kaneshiro, K.; Oliver, B. Re-annotation of eight *Drosophila* genomes. *Life Sci Alliance* **2018**, *1*, e201800156, doi:10.26508/lsa.201800156.
3. Carvalho, A.B.; Clark, A.G. Efficient identification of Y chromosome sequences in the human and *Drosophila* genomes. *Genome Res.* **2013**, *23*, 1894-1907, doi:10.1101/gr.156034.113.
4. Carvalho, A.B.; Lazzaro, B.P.; Clark, A.G. Y chromosomal fertility factors *kl-2* and *kl-3* of *Drosophila melanogaster* encode dynein heavy chain polypeptides. *Proc Natl Acad Sci U S A* **2000**, *97*, 13239-13244, doi:10.1073/pnas.230438397.
5. Belote, J.; Zhong, L. Duplicated proteasome subunit genes in Drosophila and their roles in spermatogenesis. *Heredity* **2009**, *103*, 23-31, doi:10.1038/hdy.2009.23.
6. Krsticevic, F.J.; Schrago, C.G.; Carvalho, A.B. Long-read single molecule sequencing to resolve tandem gene copies: The *Mst77Y* region on the *Drosophila melanogaster* Y chromosome. *G3* **2015**, *5*, 1145-1150, doi:10.1534/g3.115.017277.
7. Koerich, L.B.; Wang, X.; Clark, A.G.; Carvalho, A.B. Low conservation of gene content in the *Drosophila* Y chromosome. *Nature* **2008**, *456*, 949-951, doi:10.1038/nature07463.
8. Carvalho, A.B.; Clark, A.G. Y chromosome of *D. pseudoobscura* is not homologous to the ancestral *Drosophila* Y. *Science* **2005**, *307*, 108-110, doi:10.1126/science.1101675.
9. Mahajan, S.; Wei, K.H.; Nalley, M.J.; Gibilisco, L.; Bachtrog, D. De novo assembly of a young Drosophila Y chromosome using single-molecule sequencing and chromatin conformation capture. *PLoS Biol* **2018**, *16*, e2006348, doi:10.1371/journal.pbio.2006348.
10. Tamura, K.; Subramanian, S.; Kumar, S. Temporal patterns of fruit fly (*Drosophila*) evolution revealed by mutation clocks *Mol Biol Evol* **2004**, *21*, 36-44, doi:10.1093/molbev/msg236.

11. R Core Team. *R: A language and environment for statistical computing*, R Foundation for Statistical Computing: 2021.
12. Larkin, A.; Marygold, S.J.; Antonazzo, G.; Attrill, H.; Dos Santos, G.; Garapati, P.V.; Goodman, J.L.; Gramates, L.S.; Millburn, G.; Strelets, V.B.; et al. FlyBase: updates to the *Drosophila melanogaster* knowledge base. *Nucleic Acids Res* **2021**, *49*, D899-D907, doi:10.1093/nar/gkaa1026.
13. Kim, B.Y.; Wang, J.R.; Miller, D.E.; Barmina, O.; Delaney, E.; Thompson, A.; Comeault, A.A.; Peede, D.; D'Agostino, E.R.; Pelaez, J.; et al. Highly contiguous assemblies of 101 drosophilid genomes. *Elife* **2021**, *10*, doi:10.7554/eLife.66405.
14. Tamura, K.; Stecher, G.; Kumar, S. MEGA11: molecular evolutionary genetics analysis version 11. *Mol Biol Evol* **2021**, *38*, 3022-3027.
